# A conceptual framework on determinants of the integrated tuberculosis control model implementation in China

**DOI:** 10.3389/fmed.2024.1407131

**Published:** 2024-08-21

**Authors:** Xi Chen, Jiani Zhou, Quan Yuan, Chunji Huang, Ying Li

**Affiliations:** ^1^Department of Social Medicine and Health Service Management, College of Preventive Medicine, Army Medical University (Third Military Medical University), Chongqing, China; ^2^Army Medical University (Third Military Medical University), Chongqing, China

**Keywords:** tuberculosis, determinants, integrated tuberculosis control model, public service, conceptual framework

## Abstract

Improving the provision of tuberculosis (TB) care is both urgent and imperative to achieve the goals outlined in the End TB Strategy. China has initiated the integrated TB control model to enhance the quality of TB care Since 2012. Despite these efforts, the integrated TB control health system encounters numerous challenges in delivering effective TB care. The factors influencing TB care provision are intricate, and a conceptual framework to comprehend these potential determinants is currently lacking. To bridge this gap, this article proposed a conceptual framework that was developed through insights from the fields of both public management and health services, adjustment of PRISM model and elements, reference to the blocks of health system and reference to the framework of outcome indicators in implementation research. This conceptual framework included 4 modules which can be coherently and logically deduced, offered a multi-perspective understanding of the determinants to TB care, and hypothesized that the TB control services provided by the integrated TB control model is a public service and must be “patient-centered”; determinants of the integrated TB control model implementation can be divided into seven domains; the evaluation of the integrated TB control model implementation covers implementation outcomes and service outcomes. This framework offers the potential to guide empirical investigations, aiding in the understanding and identification of determinants, including barriers and facilitators, associated with the implementation of the integrated TB control health model. Furthermore, it serves as a valuable tool for developing interventions that address system-level barriers, drawing insights from the realms of public management and health services.

## 1 Introduction

Tuberculosis (TB) continues to be a major infectious disease threatening global health. Prior to the COVID-19 pandemic, TB was the leading cause of death from a single infectious agent and the 13th cause of death worldwide (1). In 2021, there were 6.4 million new TB cases reported globally, with ~1.6 million deaths due to TB (1). In recent years, remarkable achievements have been made in the global fight against TB until 2019 through the advocacy of World Health Organization (WHO) and concerted efforts worldwide. However, the COVID-19 pandemic has reversed the progress (1, 2), further compounding the challenges faced in the global TB control efforts. Accounting for the third highest number of incident TB cases in the world (7.4%) (1), China has been taking proactive measures to in responses to WHO's endeavor to control TB epidemic. Between 2015 and 2019, China reached an average annual decline of 3.4% in notified TB incidence rates (3). However, the reductions in both incidence and mortality rates of TB, especially multidrug-resistant TB (MDR-TB), have fallen below expectations in China (4). In addition, four provinces/autonomous regions in West China still reported TB incidence rates exceeding 100/100 000 (5).

To effectively tackle the challenges of TB prevention and control and to achieve the goal of ending TB, the Chinese government has issued a series of policies and measures. A pivotal one is the 12th Five-Year Plan for National Tuberculosis Prevention and Control (2011–2015) (6). This policy required a gradual shift from the center for disease control and prevention (CDC)-based vertical TB control model to the integrated TB control model nationwide. This novel model is an integrated horizontal healthcare system which requires the collaboration among CDCs, TB-designated hospitals and primary healthcare (PHC) sectors. Under the integrated TB control model, the CDCs monitor TB epidemic and coordinate TB control service provided by TB-designated hospitals and primary healthcare (PHC) sectors; the responsibility of TB-designated hospitals primarily lies in the diagnosis, treatment and registration of TB patients; and PHC sectors are in charge of referrals of presumed TB patients and follow-up of patients diagnosed with TB (7, 8). This integrated TB control model advocates for the establishment of an active communication and cooperation mechanism among these health sectors to jointly deliver “patient-centered” high-quality TB control services (9, 10), with the aim of improving the effectiveness and efficiency of TB control programs and making it more affordable and sustainable (11).

The transition to this TB control model marks a significant milestone in China's fight against TB (12). Over the past decade, the integrated TB control model has been scaled up nationwide, but the TB burden is still heavy in China (1). To achieve the goals in the End TB Strategy, China faces great challenge (11) and needs to intensify its efforts to substantially reduce the TB incidence rate from 55/100,000 in 2021 to 33/100,000 by 2025 (7, 13). Therefore, it is urgent to identify the factors that hinder the effectiveness and efficiency of the integrated TB model and propose evidence-informed strategies to improve TB care.

In the past decade, some studies conducted evaluation on integrated TB control service with TB case notification and mortality rates, satisfaction of healthcare workers' (HCWs) TB management, tuberculosis treatment management services (TTMSs) delivery rate, adherence rates of patients to treatment and human resources allocation (5, 8, 14–20). A study from Guangxi Province in China outlined an indicators framework designed to assess the performance of TB control in primary healthcare settings from discovery, report, registration of patients and quality control (21). Actually, an intervention or program implementation cannot be effective if it is not implemented well, and it is important to know whether the intervention or program was deployed correctly (implementation success) and monitor implementation outcomes which serve as the necessary preconditions for desired change in service and treatment outcomes (22). However, previous studies only used indicators either on treatment outcomes or some aspects of TB program implementation failing to comprehensively monitor or evaluate the implementation of the integrated TB control model, such as the acceptance among patients or TB HCWs, the feasibility in specific settings and the equity of TB care under this TB control model (22).

Few determinants, including inadequate resources and incentives, insufficient training, poor cross-sectional coordination, low socioeconomic status, poor health literacy and TB-related social stigma, have been identified in related to integrated TB control service delivery (15–17). The Practical, Robust Implementation and Sustainability Model (PRISM) indicated that the improvement of a healthcare program or intervention implementation should identify factors from the design of the program or intervention, the external environment, the implementation and sustainability infrastructure, and characteristics of recipients (23). Thus, existing findings about determinants to integrated TB control service seems inadequate due to the lack of the guidance from theories, conceptual models or frameworks. Theories, as interrelated and structured concepts and propositions, possess the ability to explain and predict real-world phenomena by providing conceptual frameworks and illustrating intricate relationships between various features or variables (24, 25).

Furthermore, over the past decade, studies on TB control services utilizing theoretical or conceptual models or frameworks have been rarely reported. While several international studies developed conceptual frameworks as guidance for assessing TB-related interventions (26–28), research contexts based on public-private health institution collaboration may make them inapplicable for China's integrated TB control model currently. In China, one study leveraged the structure-process-outcome model to craft a campus TB control, analyzing the current status of TB control in campus and proposed several strategic interventions for improvement (29), while the other devised a multifaceted intervention with the guidance of the Health Action Process Approach (HAPA) model to enhance the self-management precursor among the elderly with TB (30). These efforts on TB control in China lack design of strategies improving the integrated TB control model implementation. Moreover, TB care under the integrated TB control model is a public product of the public policy. And Stop TB Strategy proposed by WHO recommended that patient-centered care and supervision must be carried out in a context-specific and patient-sensitive manner in order to improve patient adherence to TB treatment (31). Therefore, public nature and patient-centered approaches should be taken into account in identifying determinants of TB program and developing relevant interventions or implementation strategies.

Consequently, a systematical theoretical model or framework that guides the comprehensive understanding of the whole integrated TB control model remains unexplored. This study aimed at addressing this gap by proposing a preliminary conceptual framework that can guide comprehensive evaluation on, identification of potential determinants related to and design of effective implementation strategies toward the integrated TB control model implementation, by extensively referring to the theories, concepts, models or frameworks from previous studies and combining them with the distinctive features of the integrated TB control model. The framework will serve as a starting point for the development of subsequent frameworks aimed at comprehending TB control system implementation in diverse settings and countries.

## 2 Method

### 2.1 Search strategy

After analyzing the characteristics of the integrated TB control model and its service delivery, this study rests on the following assumptions for choosing the theories:

a. The integrated TB control model is actually a health system and provide TB care as a public service and a public product. Consequently, theories related to health system, public service and public products could be used as reference to potentially guide the understanding of TB care.
b. The ultimate aim of the integrated TB control model is to end the TB epidemic and promote population health. So it serves TB patients or the public. Consequently, it is necessary to integrated the concepts of “people-oriented” and “patient-centered” into the integrated TB control model operation and service provided by it.

Following these considerations, a literature review was conducted across PubMed, Cochrane, EMBASE, and Web of Science databases using MeSH terms and relevant keywords, such as “public administration,” “public service,” “public health,” “health service research,” “health intervention,” “health program,” “patient-centered,” “infectious diseases,” “infectious disease control,” and “integrated control,” to identify eligible articles published from 2000 to 2023.

### 2.2 Inclusion and exclusion criteria

The inclusion criteria included:

1. Type of articles: original research, review, perspective, hypothesis and theory, general commentary, opinion, editorial;
2. Content of articles: research content was related to development of, description of or discussion about theories, concepts, models, or frameworks;
3. Research field of articles: articles which were published in the fields of public administration, public service, public health and infectious disease prevention and treatment were included.

The exclusion criteria were: (1) articles including methods, clinical trial, case report were excluded; (2) articles about basic researches in natural sciences; (3) articles whose findings came from newspapers or conference papers; (4) articles without full texts.

### 2.3 Selection of referring theories and their enlightenment

Based on considerations and literature review described above, we selected relevant theories, concepts, models, or frameworks, and subsequently delved into their implications for enhancing our understanding of the integrated TB control model.

#### 2.3.1 New Public Service Theory

**Figure 1:**
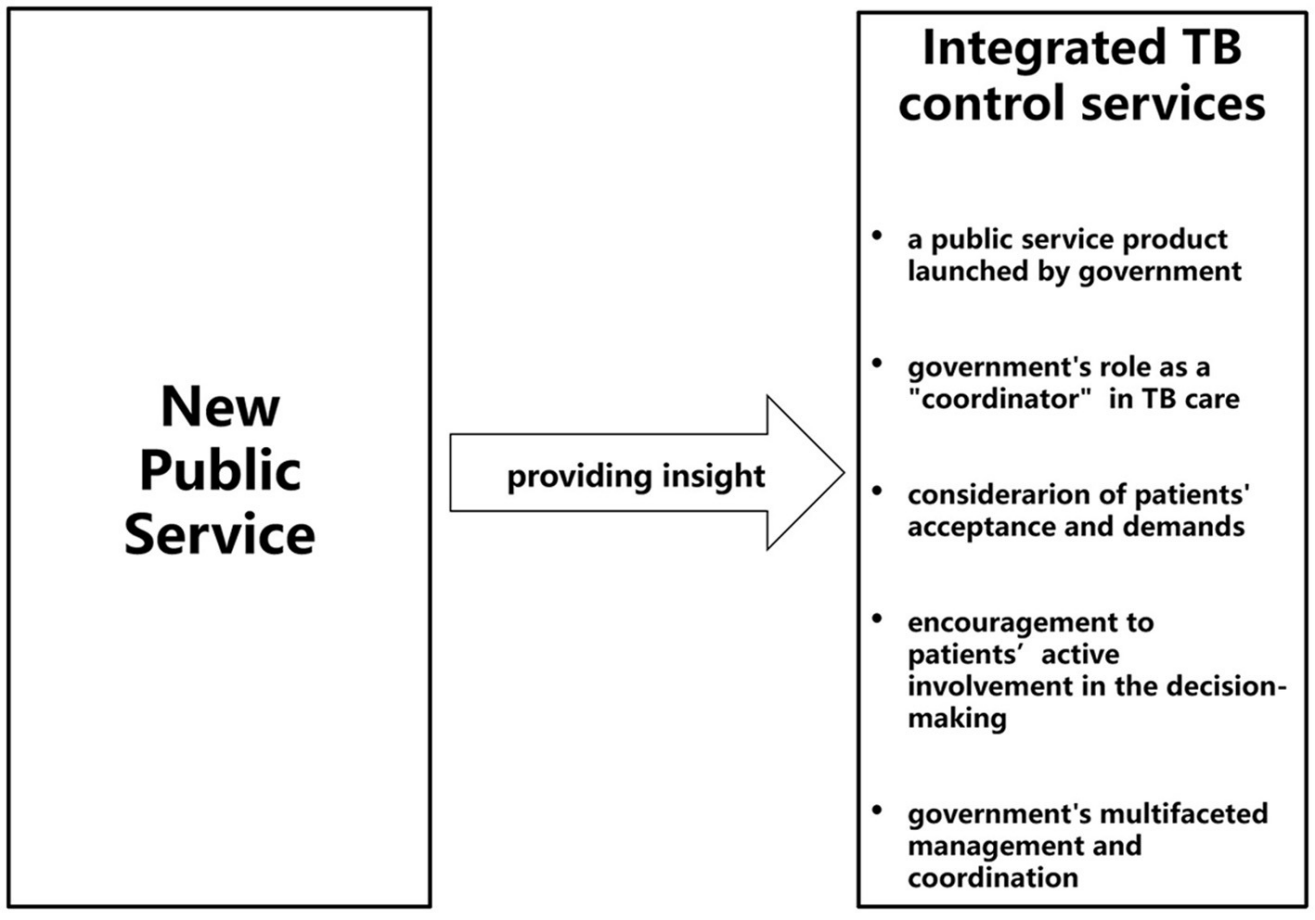
Integrated TB control services under insight from the New Public Service Theory. This figure presents that under insight from the New Public Service Theory, the integrated TB control services provided by the integrated TB control model can be viewed as one kind of public service product, in which government play multiple roles in TB care provision.

The New Public Service Theory was developed on the basis of incorporating the important value of the New Public Management Theory in improving public management practice while discarding its inherent defects (32, 33). It is a novel theory that prioritizes democratic values and public interest, making it more suitable for modern public society and public management practice (34, 35). The main viewpoints of the New Public Service Theory contain: (1) Serve, not steer; (2) Pursue public interests as the goal; (3) Think strategically and act democratically; (4) Serve citizens, not customers; (5) Responsibility is not single, but diversified; (6) Value persons, not just productivity; (7) Value citizen's rights and public affairs. Under the insight from the New Public Service Theory, TB control services in the integrated TB control model should be identified as a kind of public service (see Figure 1).

Firstly, the New Public Service Theory posits that the ultimate goal and the primary manifestation of public management measures undertaken by governmental administrative agencies, should aim to realize the public interests of the majority of citizens (34, 35). The integrated TB control model, as a public policy implemented by these agencies, should similarly strive to achieve the public interest of ending the TB epidemic and promoting the population health. Hence, under insight from the New Public Service Theory, integrated TB control services can be conceptualized as a tailored public service product devised by the government for TB patients. This theory also advocates for the government to act as a mediator and facilitator in actualizing public interests, fostering dialogue and ensuring effective collaboration among diverse departments (35, 36). In this context, the government's role as a “coordinator” is crucial for establishing a seamless and cohesive cooperation mechanism within the integrated TB control model.

Secondly, the New Public Service Theory emphasizes that the government should prioritize meeting the genuine demands and interests of service recipients, thereby enhancing service responsiveness and fostering active participation of patients in the realization of public interest (32, 35, 37). Analogously, under the context of integrated TB control services, the development and adjustment of treatment and management plans ought to comprehensively incorporate the patients' preferences and demands and encourage their proactive involvement in the decision-making processes. This necessitates the government respecting patients' demands and interests, and empowering them to transit from being mere “passive service object” to “active participant” in shaping their own healthcare journey (33, 36).

Thirdly, the New Public Service Theory posits that within the public management system, governmental responsibility transcends merely issuing and enforcing policies in a unilateral and mandatory manner (37). Instead, it emphasizes the need for public managers to foster a “service-oriented” governmental approach (34, 35, 38). TB prevention and control is a long-term and complex process involving numerous stakeholders and influencing factors. As the top-level planner for TB control services strategy and societal “servant,” the government must assume a multifaceted role in management and coordination to deal with the intricate interplay of societal factors. To ensure the effective implementation and sustainable development of integrated TB control services, the government's mandate extends beyond policy formulation and funding allocation to conflict resolution between competing interests and workstreams, taking social, cultural, environmental, democratic, ethical and more factors into account. In the allocation of healthcare resources, the government should undertake diversified responsibilities of comprehensive regulation and multilateral coordination (32, 35, 37).

Therefore, the New Public Service Theory could be applied to build a conceptual framework for comprehensively understanding the potential determinants related to the integrated TB control model implementation. However, it is important to acknowledge that although this theory provides macro-level guidance and analysis, it lacks specific metrics or frameworks tailored to TB control service research. For instance, in what aspects should the public attributes of TB control services be considered? In what aspects does the government's comprehensive regulation reflect and how does it affect TB control services? Thus, we propose that it is necessary to further deduce or combine the New Public Service Theory with other theories, concepts, models or frameworks for theoretical supplement and improvement when applying it to guide research on TB control services.

#### 2.3.2 Patient-centered care

The widely accepted definition of Patient-Centered Care (PCC) in the field of health research is proposed by the Institute for Patient and Family-Centered Care (IPFCC), encompassing four core concepts: maintenance of patients' dignity and respect for patients, information sharing, participation of patients and families, and collaboration among patients, families, medical staffs, and healthcare administrators (39, 40). Moreover, Gerteis et al. (41) identified six dimensions of PCC: (1) respect for patients' values, preferences, and expressed needs; (2) coordination and integration of care; (3) information, communication, and education; (4) physical comfort; (5) emotional support—relieving fear and anxiety; and (6) involvement of family and friends. PCC aims to customize healthcare services to meet the specific needs and circumstances of each individual patient. It advocates a fundamental shift from a one-size-fits-all model to a responsive mode that adjusts services to meet the person, rather than expecting the person to conform to services. This paradigm enhances care experiences, improves health outcomes for patients and their families, and boosts the satisfaction of clinical staffs, thereby motivating service providers to allocate resources more effectively (39, 42). Several studies suggest that PCC should first view patients as a whole, and healthcare services should simultaneously consider and address the patients' multiple demands, the environment they live in, and their physical, cognitive, and psychological functions. Secondly, the provision of PCC should be personalized, taking into account the patients' unique preferences, personality, and concerns about health status. Finally, PCC should respect and empower patients as active consumers, and encourage their informed decision-making and self-management (43, 44).

**Figure 2:**
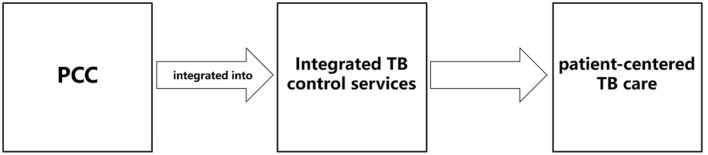
Integrated TB control services integrated with patient-centered care concept. This figure shows the conceptual guidance of the PCC concept to integrated TB control services which requires the delivery of patient-centered TB care provided by the integrated TB control model.

WHO has highlighted the PCC as an important pillar of the End TB Strategy, emphasizing that providing patient-centered services and support, which can sense and respond to the patients' educational, emotional and physical demands, is the foundation of the global TB strategy (45). The End TB Strategy defines PCC as the recognition and resolution of patients' demands and expectations, advocating for its design and implementation to be contextually sensitive and tailored. Some researchers argued that the goal of patient-centered TB healthcare services extend beyond only addressing challenges related to disease treatment (46), or improving policies and planning, but also recognizing the diverse demands of TB patients when providing comprehensive TB prevention, care and treatment (47). In recent years, following the initiative of WHO End TB Strategy, PCC has attracted increasing attentions on TB prevention and control, with a number of studies reporting the incorporation of PCC concepts in the development, application and improvement of TB control intervention strategies (48–50). Therefore, in the context of the integrated TB control model in China which responses actively to the global strategy, the integration of PCC into the delivery of TB control services is imperative (see Figure 2).

Nevertheless, it is crucial to acknowledge the limitations of PCC concept as a purely conceptual guidance and supplements from the perspective of safeguarding patients' key interests, which is inadequate in the establishing the conceptual framework related to TB control services. This conceptual guidance does not directly offer detailed paths for conducting research on TB control services.

#### 2.3.3 PRISM model

PRISM was developed by Feldstein and Glasgow (23) and integrated theoretical concepts of quality improvement, chronic care, the diffusion of innovation, measures of effectiveness of translating research into practice, and classic theoretical models like PRECEDE/PROCEED and RE-AIM, to establish a comprehensive framework for assessing the potential influencing factors and their interactions in the implementation of health programs or intervention measures. At present, the PRISM model is applied in the evaluation, development, implementation, sustainable development and promotion of health intervention programs (51). It presents a comprehensive list of factors (39 in total) associated with health intervention programs and divides them into four domains (23). A previous study successfully used the adapted PRISM to identify the barriers to TTMSs in PHC sectors in China (17).

**Figure 3:**
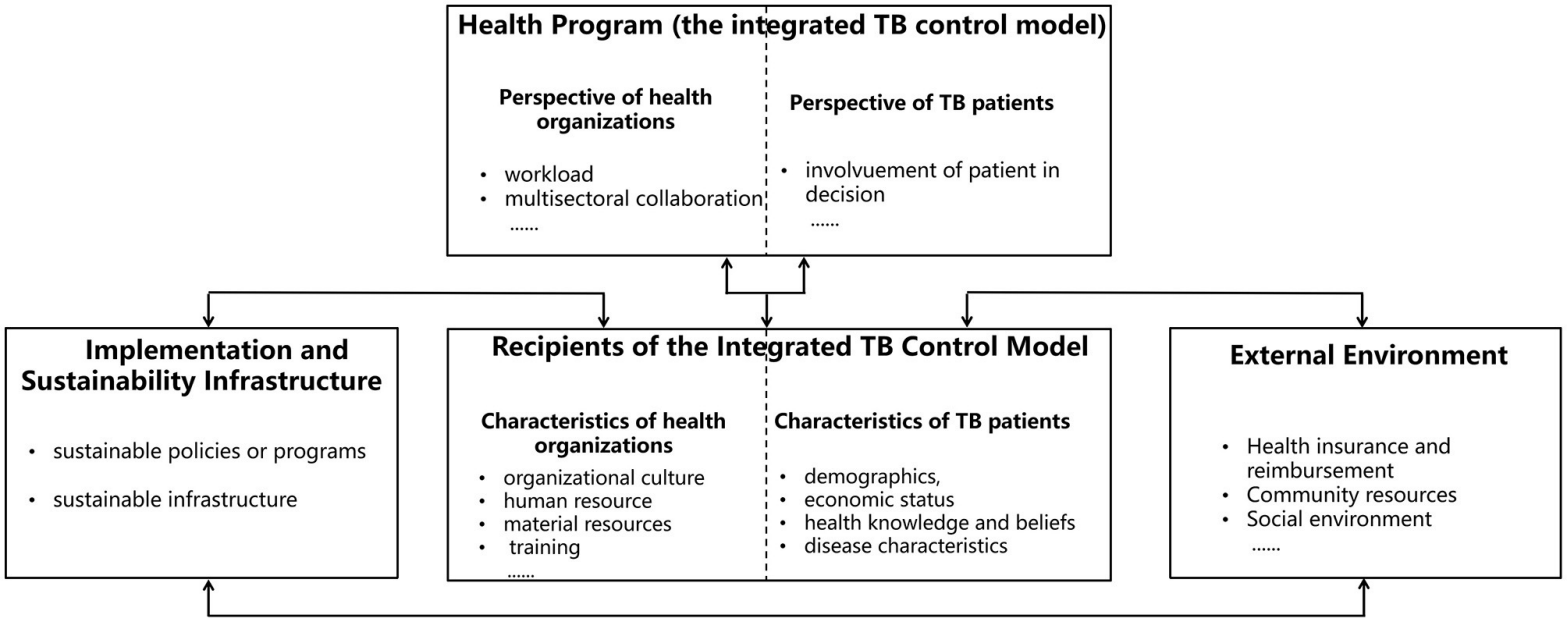
Determinants related to the integrated TB control model implementation guided by the PRISM model. This figure indicates the understanding and analysis of determinants of the integrated TB control model implementation. These determinants can be divided into four domains under the guidance of the PRISM model including health program, recipients of the integrated TB control model, implementation and sustainability infrastructure, and external environment.

Offering a comprehensive and universally applicable framework for health program research, the PRISM model deserve application in understanding the integrated TB control model (see Figure 3). Firstly, the PRISM model proposes that determinants associated with health programs should take into account the perspective and characteristic of health organizations, which encompass not only the practitioners of health programs (frontline healthcare staffs), but also top-level leaders and mid-level managers (23). From the perspective of health organizations, both workload and the requirement for multisectoral collaboration may influence the implementation of TB control services. Furthermore, according to the PRISM model, health organizations and their members serve as health regulators or managed entities, whose culture, human resource and material resources, training and others in the TB control model may affect the delivery of TB control services. Secondly, within the perspective of PRISM model, the integrated TB control model provides TB control health program and involves multiple stakeholders that go beyond service providers and service recipients, and its external environment as important associated factors. Thirdly, PRISM model highlights the pivotal role of the patient's perspective and characteristics, such as economic status, health knowledge and beliefs, and disease characteristics in health program implementation (23), which required reduction of patients financial burden, involvement of patient in decision on disease prevention and treatment, and consistency with PCC concept of the integrated TB model. Besides, the PRISM model underscores the significance of robust implementation and sustainability infrastructure and encourages policymakers to strengthen and improve pertinent policies, programs, and infrastructure to ensure sustainability of the integrated TB control services (11).

However, some influencing factors outlined in the PRISM model are not applicable to the in-depth understanding of TB control services or the analysis of its related determinants. Furthermore, this model lacks the encapsulation of certain key features specific to TB control services, such as their public attributes, within its framework and factor list. Therefore, when employing the PRISM model as a guidance to comprehend and analyze the integrated TB control services and establish a conceptual framework, it is necessary to either deduce or integrate it with other theories, concepts, models or frameworks to enhance and refine the applicability to certain context.

#### 2.3.4 Conceptual model of implementation research

Implementation research is the scientific research process that systematically integrates research findings and evidence-based intervention programs into routine practice to improve the quality and effectiveness of health services and healthcare (52). Theoretical frameworks play a vital role in facilitating the comprehension of implementation determinants, conceptualizing and measuring implementation outcomes, and fostering the effective dissemination of research findings (53, 54). Proctor et al. (55) proposed a heuristic conceptual model of implementation research by integrating multiple classic theoretical models (56–62). They further optimized the framework of outcome indicators in implementation research by integrating several theories and concepts specific to implementation research (22, 63–66), resulting in a robust set of eight conceptually distinct implementation outcomes indicators. Classifying and defining these indicators explicitly, Proctor et al. crafted a framework for implementation researchers to evaluate implementation strategies. Currently, this framework has been successfully applied to diverse healthcare domains, including clinical trial management, patient quality of life improvement, obstetric nursing, AIDS prevention and control, and community health services (67–71).

**Figure 4:**
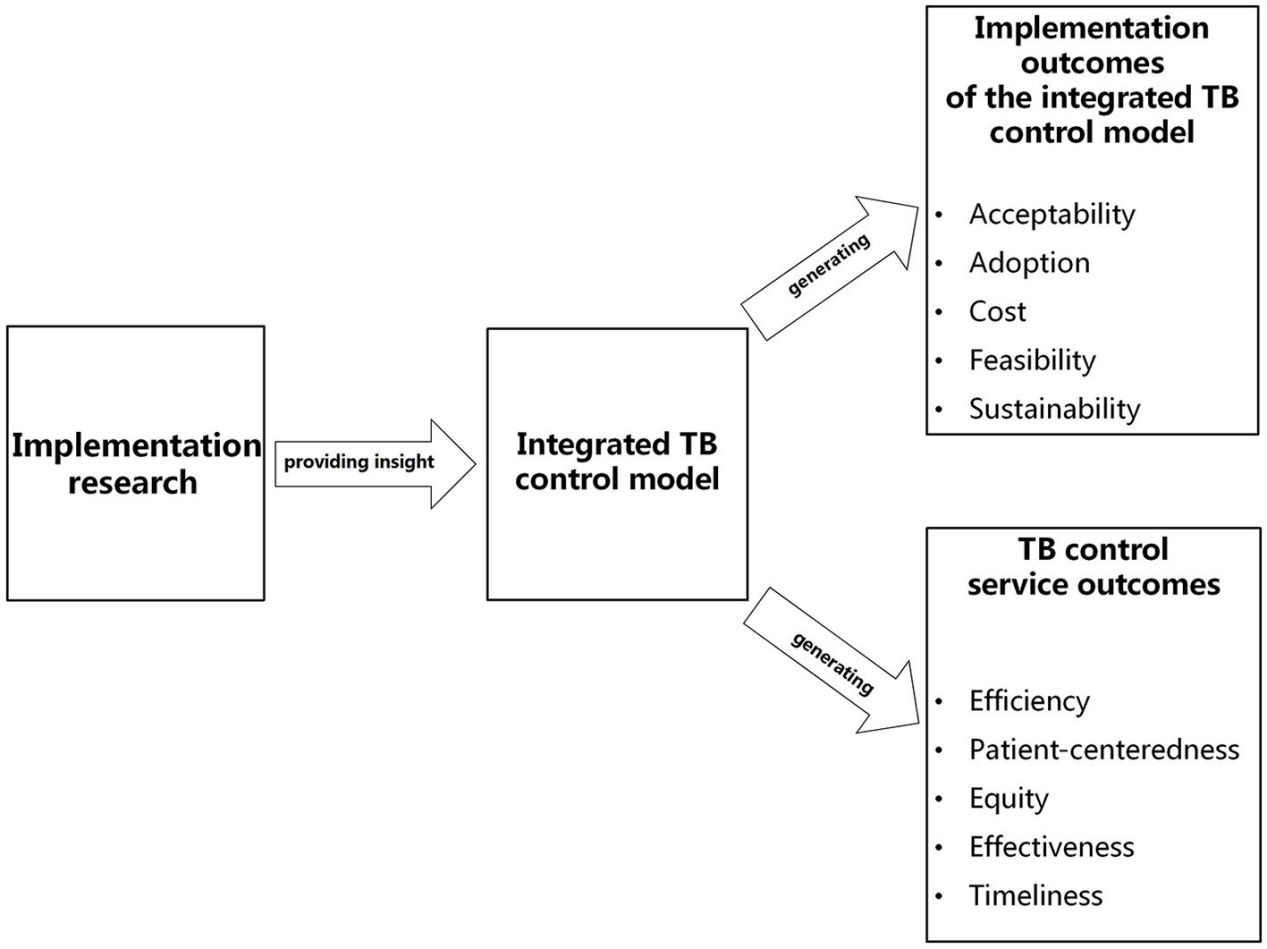
Outcomes in the integrated TB control model implementation under insight from implementation research. This figure illustrates that with the insight from implementation research theory and adoption of the framework of outcome indicators in implementation research, the evaluation of the integrated TB control model implementation should cover implementation outcomes and service outcomes, both of which may include five evaluation dimensions.

Given the long-standing effectiveness and nationwide implementation of the integrated TB control model, evaluating its implementation process and outcomes has become important. We posit that it is both appropriate and feasible to assess the implementation of the integrated TB control model under the insight from implementation research theory and the adoption of the framework of outcome indicators in implementation research (implementation outcomes, service outcomes, patient outcomes). Implementation outcomes of the integrated TB control model may include acceptability, adoption, cost, feasibility and sustainability; TB control service outcomes would include efficiency, patient-centeredness, equity, effectiveness and timeliness (see Figure 4).

## 3 Results

### 3.1 Establishment and interpretation of the conceptual framework

**Figure 5:**
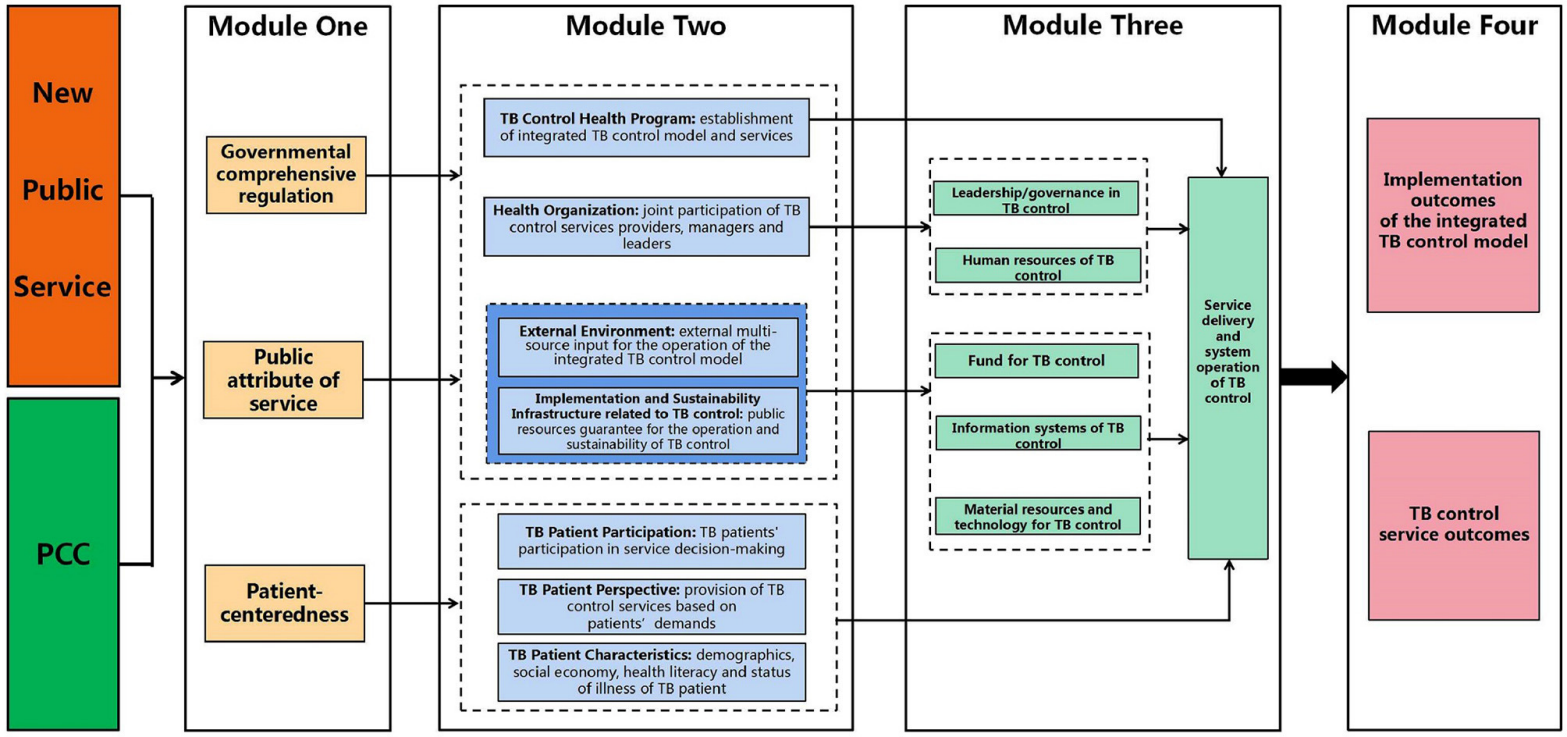
Conceptual framework on determinants of the integrated TB control model implementation. This figure presents that the establishment of conceptual framework on determinants of the integrated TB control model implementation, including four modules, under the guidance and integration of the New Public Service Theory, PCC concept, the PRISM model and the framework of outcome indicators in implementation research.

After meticulously analyzing pertinent theories, concepts, and models (Figures 1–4), we integrated these theoretical guidance with distinct features of the integrated TB control model implementation. Thus, a preliminary conceptual framework on the determinants of integrated TB control model implementation was developed (see Figure 5).

### 3.2 Modules of the conceptual framework

*Module One: Macro concepts through the integration of the New Public Service Theory and the concept of PCC*.

To enhance the comprehension and application of abstract theories and concepts, we refined and integrated seven main viewpoints of the New Public Service Theory. Specifically, the notion of “serve, not steer” was integrated with that of “pursuing public interests as the goal,” yielding the concept of “*public attribute of service*.” Additionally, we consolidated the concepts of “think strategically and act democratically,” “serve citizens, not customers,” “value persons, not just productivity,” and “value citizen's rights and public affairs” into the concept of “*value patients' demands and participation*.” Furthermore, the idea of “responsibility is not single, but diversified” was integrated into the concept of “*comprehensive regulatory role of government*.” Then, we refined and integrated the concept of PCC into six aspects including *respect for patients' willingness and demands, coordination and integration of healthcare services, emotional support, physical comfort, care for patient and participation of social relationships*.

Given that both the New Public Service Theory and PCC are conceptual, we decided to synthesize them at the conceptual level and thus formed Module One. This module encompasses three concepts: governmental comprehensive regulation, public attribute of service and patient-centeredness. The first two concepts derived primarily from the main viewpoints of the New Public Service Theory. However, the concept of “*value patients' demands and participation*” within the New Public Service Theory appears to be not comprehensive and insufficient for considering and understanding patients' interests. On the other hand, the six concepts contained in PCC are well complementary and improved conceptually. Therefore, in Module One, we preferred to form the third concept, patient-centeredness, on the basis of the concepts of PCC. Consequently, the Module One potentially offers macro-level conceptual guidance for comprehending and analyzing the influencing factors related to the integrated TB control services.

*Module Two: Determinants of the integrated TB control model implementation through adjustment of PRISM model and elements*.

The PRISM model presents an extensive list of reference elements (39 in four domains) (23). However, these elements are not completely applicable for the identification of determinants related to specific health programs (such as TB prevention and control). Hence, in this study, we modified and adjusted the PRISM model and the determinants it provides as necessary. Firstly, we adopted the four domains in the PRISM model and selected and improved the determinants within each domain by considering the characteristics of TB control services. Secondly, we summarized the determinants within the four domains, and further classified and integrated these domains according to the three concepts in Module One. Notably, we extracted and integrated the perspectives and characteristics of TB patients from the domains under the perspective of patient-centeredness. Ultimately, Module Two was constructed and generated with 7 domains–the module of determinants of the integrated TB control model implementation.

*Module Three: Integrated TB control health system with reference to the blocks of health system*.

As mentioned above, the establishment of the integrated TB control model is built upon the existing organizational structure of national and local health system, which includes the Health Committee, CDC, general hospitals, and PHC sectors (72, 73). Within this system, national and local governments mandate TB control services, the health administrative department (usually the local health committee) coordinates and supervises policy implementation, and the three main TB control institutions provide TB control services (72). Therefore, it is both feasible and necessary to comprehend the integrated TB control model from the perspective of the health system. However, certain characteristics of TB control, such as the long treatment cycle and strict requirements for medical professional competence, make the TB control system different from the routine health service system in practical operation. We referred to the six blocks of health system proposed by WHO (73) and integrated them with the characteristics of TB control services to develop the blocks of the integrated TB control health system. These blocks include leadership/governance in, human resources of, fund for, information systems of, material resources and technology for, service delivery and system operation of TB control. We then classified and integrated these blocks according to the hypothetical potential influence of determinants within the seven domains in Module Two, resulting in the construction of Module Three.

*Module Four: TB control outcomes according to the framework of outcome indicators in implementation research*.

In the Module Four, we initially understood and cognized the integrated TB control model and its service delivery under the insight from implementation research. Subsequently, we referred to the framework of outcome indicators in implementation research to conduct the multi-dimensional analysis of outcomes resulting from TB control services during different stages of implementation, which includes the implementation outcomes of the integrated TB control model and TB control service outcomes. Thus, we established the module of the integrated TB control outcomes. Notably, we did not integrate the patient outcomes into Module Four since some indicators in this dimension, such as morbidity, satisfaction, social function, could be affected by many contextual factors not related to TB control services, making these outcomes improper to be used to directly evaluate the effect of the integrated TB control model implementation and delivery of TB control services. Moreover, when applying Module Four to TB control services research, it is necessary to expand the dimensions of evaluation from the framework of outcome indicators in implementation research, and to study and develop practical indicators that align with the characteristics of the integrated TB control services.

### 3.3 The internal logic and mechanisms of the conceptual framework

Based on the conceptual framework on the determinants of the integrated TB control model implementation, we analyzed the internal logic and mechanism, and proposed hypothesis.

a. Module One provides three macro conceptual perspectives based on the New Public Service Theory and PCC concept. These perspectives guide the re-understanding and analysis of determinants related to the integrated TB control model implementation in Module Two. Under the insight from both *governmental comprehensive regulation* and *public attribute of service*, it is necessary to consider 4 sets of determinants: establishment of integrated TB control model and services in TB Control Health Program domain, joint participation of TB control services providers, managers and leaders in Health Organization domain, external multi-source input for the operation of the integrated TB control model in External Environment domain, and public resources guarantee for the operation and sustainability of TB control in Implementation and Sustainability Infrastructure Related to TB Control domain. Additionally, under the perspective of *patient-centeredness*, three sets of determinants are identified: TB patients' participation in service decision-making in TB Patient Participation domain, provision of TB control services based on patients' demands in TB Patient Perspective domain, and demographics, social economy, health literacy and status of illness of TB patient in the TB Patient Characteristics domain.
b. The determinants in each domain of Module Two are potentially relevant to the blocks of the integrated TB control health system in Module Three respectively. Determinants in TB Control Health Program domain may affect service delivery and system operation of TB control in Module Three; those in Health Organization domain indirectly influence service delivery and system operation of TB control by potentially acting on the leadership/governance in TB control and human resources of TB control; determinants in External Environment domain and Implementation and Sustainability Infrastructure Related to TB Control domain impact the fund for TB control, information systems of TB control, and material resources and technology for TB control, which collectively affect service delivery and system operation of TB control; determinants from domains of the patient level (TB Patient Participation, Perspective, and Characteristics) directly influence service delivery and system operation of TB control.
c. The determinants in Module Two potentially affect TB control outcomes (Module Four) by acting on Module Three. Notably, the mechanism by which the Module Three, as a mediating variable, influences each TB control outcome, which needs further clarification through path analysis based on abundant empirical studies.

We then proposed three hypotheses as follows:

- Hypothesis 1: the TB control services provided by the integrated TB control model need comprehensive governmental regulation, should possess the attributes of public services, and must be “patient-centered.”
- Hypothesis 2: determinants of the integrated TB control model implementation can be divided into seven domains including TB control health program, health organization, external environment, implementation and sustainability infrastructure related to TB control, and TB patient participation, TB patient perspective and TB patient characteristics.

2.1 The determinants in TB control Health Program domain should focus on establishment of integrated TB control model and services;

2.2 The determinants in Health Organization domain should focus on the joint participation of all providers in integrated TB control services;

2.3 The determinants in External Environment domain should focus on external multi-source input for the operation of the integrated TB control model;

2.4 The determinants in Implementation and Sustainability Infrastructure Related to TB Control domain should focus on public resources guarantee for the operation and sustainability of TB control;

2.5 The determinants in TB Patient Participation domain should focus on their participation in service decision-making;

2.6 The determinants in TB Patient Perspective domain should focus on provision of TB control services based on patients' demands;

2.7 The determinants in TB patient Characteristics domain should focus on demographics, social economy, health literacy and status of illness of TB patient.

- Hypothesis 3: the evaluation of implementation of the integrated TB control model needs analyze implementation outcomes and service outcomes.

In summary, this conceptual framework can guide the collection and analysis of evidence on determinants related to the integrated TB control model implementation and its outcomes. Such research evidence further provides a key basis for designing and implementing targeted, context-specific intervention strategies to improve the quality of TB control services.

## 4 Discussion

There is currently a lack of theoretical model or framework to fully understand and analyze the integrated TB control model implementation. This article integrated or adjusted four theories, concepts and models based on the characteristics of the integrated TB control model, and finally proposed a framework at system level and insight from public health service. This framework have shown how to understand the integrated TB control model and would contribute to assist empirical research related to the integrated TB control model, including evaluation of TB control service implementation, identification of determinants, development of intervention strategy.

### 4.1 Providing a multi-perspective understanding of the integrated TB control model

Module One integrating the New Public Service Theory and PCC provides a reasonable and appropriate macro insight to understand TB control services. The Stop TB action plan (2019–2022) clearly emphasized government leadership and cooperation of the whole society in TB control in China (74). The complexity of TB control inevitably requires cross-sector cooperation of multiple governmental departments at the leadership level, such as the Education Bureau, the Finance Bureau, and the Health Insurance Bureau, not just the Health Committee.

As one of the key basic public health services programs available to all residents in China (75), TB healthcare is delivered by public health facilities in China with resources provided almost entirely by the government. According to Module One, the integrated TB control model provides a public service for achieving public health goals for the whole population, including TB patients. For such a public service, it is essential to emphasize and focus on public attributes of the integrated TB control model and its products (TB service), and government's regulatory roles in assisting its operation. This framework may push researchers to transcend inherent cognition to integrated TB control service as a mere procedural task for healthcare sectors, where government decisions are implemented as per documented guidelines. It fosters an innovative and comprehensive understanding of the model's implementation from a broader macro perspective and aligns TB care more closely with public management consciousness and aspirations, which underscoring its advantages different from existing models or frameworks.

According to Module One of this framework, TB patients, as receiver of TB care from the integrated TB control model, should not be seen solely as passive recipients of TB control services. On the contrary, they have the right and need to participate in decision-making processes related to their own health interests (42, 76). In real-world context, medication, supervision management and health education are TB care that patients can directly access. The initial formation of these TB control service programs cannot be uniform and must be adjusted according to the actual situation in the long treatment period for TB. The phase/type/dosage/means of medication, the provider/means/phase/frequency/content of supervision management and health education were necessarily determined with involvement of TB patients in making these decisions (72). Therefore, it is imperative to meet the demands and willingness of TB patients when the integrated TB control model provides TB care. For example, patients' willingness to receive supervision management or their preferred means of health education. Nevertheless, this process would be challenged by poor awareness of patient-centered service delivered by HCWs and the receivers' low health literacy (17, 45), especially in resource-limited areas. Rigid delivery of health services, which are divorced from patients' demands and participation, and limited patient-centered approaches may inevitably lead to a decline in patient adherence and satisfaction to TB treatment and management (15–17, 45, 47).

The integrated TB control model is often perceived as a system, and assessing the success of this system requires observing and evaluating its short- and long-term implementation effects within a specific region (such as a province or city or county) (52, 77). Module Two of this framework assist us to understand the integrated TB control model from the perspective of health programs or interventions. And therefore, determinants of its implementation include seven domains. Notably, while characteristics of individuals and interventions, outer environment and sustainable resources were commonly identified and underlined in several classical theoretical models guiding heath service or intervention implementation, such as the PRISM and the Consolidated Framework For Implementation Research (CFIR) (23, 78), we novelly proposed “TB patient participation” as a pivotal determinant to expand conceptual perspective and form a more comprehensive framework for researchers.

*Technical Guidelines for tuberculosis Prevention and Control in China (2020)* issued by Chinese National Health Commission listed the assessment dimensions related to TB control under the integrated TB control model, which mainly assess logistic measures implementation, financial burden of TB patients, TB patients detection, TB laboratory test, TB patients management and TB control of special population (72). In this study, referring to Module Four of the framework, we assess the integrated TB control model from perspectives of the quality and sustainability of TB care rather than evaluation on completion of task related to TB control.

### 4.2 Integrating multiple theories and consequently providing multiple contributions to researches on the integrated TB control model

The conceptual framework we established is not a simple patchwork of four applicable theories, concepts and models. Instead, it represents a thoughtful integration of these theories based on an in-depth understanding of their fundamental principles, core concepts and the characteristics of the integrated TB control services. The four modules in this conceptual framework consist of guiding concepts from a macro perspective (integration of the New Public Service Theory and the PCC) as well as a reference framework for research practice (adjustment of the PRISM model and the framework of outcome indicators in implementation research). Through integration and adjustment, the cited theories, concepts and models can complement and improve each other instead of being split apart. As a result, this conceptual framework related to integrated TB control services, encompassing four modules, basically generated a set of determinants in different domains guided by macro concepts. The interconnected modules enable the conceptual framework to potentially guide each part of the entire path of empirical research (implementation evaluation, identification of determinants and development of intervention strategy) on the integrated TB control model. Specifically, the framework contributes to:

(1) Evaluation of the implementation of the integrated TB control model. By integrating the theoretical principles of implementation research and the framework of outcome indicators in implementation research as guidance, the evaluation dimensions and perspectives of implementation of the integrated TB control services can be significantly expanded, providing health policymakers and researchers with a set of reference indicators for assessing implementation outcomes and service outcomes. These indicators go beyond routine performance assessments recommended by official documents (72, 75). More importantly, such broader evaluation dimensions and perspectives may guide establishment and development of several comprehensive indicators frameworks, which hold theoretical and practical value for evaluating the integrated TB control services from initiation of implementation to the ongoing process and sustainable development. In the context of the integrated TB control model, we proposed that the evaluation of implementation outcomes may focus on acceptability, adoption, cost, feasibility, and sustainability while service outcomes should incorporate efficiency, effectiveness and equity timeliness of service and patient-centered service delivery. In an American clinical site, Jones et al. (79) adopted acceptability, adoption, and feasibility to evaluate two initiatives related to prior authorization for medications, providing evidence for local healthcare systems to improve them. By assessing changes in implementation and service outcomes, researchers from Kansas City aimed to evaluate community health improvement plans and develop implementation strategies that can quickly be integrated into practice to improve population health and health equity (69). Notably, implementation outcomes and service outcomes proposed in this study also deserve priority for comprehensive evaluation on TB care implementation under TB control systems similar, even not similar, with China's integrated TB control model in diverse contexts.
(2) Identification of determinants related to the integrated TB control model implementation. This conceptual framework adopts the domains from the PRISM model and defines the providers and receivers of integrated TB control services as top-level leaders, mid-level managers, front-line healthcare staffs and patients respectively. We adjusted and transformed the recommended elements in PRISM model based on the integrated TB model and its implementation context, ultimately clarified a unique set of determinants specific to TB control services provided by the integrated TB model. Guided by the macro concepts outlined in Module One, the purposeful classification and integration of domains assist health policymakers and researchers to take those domains into consideration while they analyze causes of different TB control outcomes because those domains decide the blocks listed in Module Three. The specific mechanisms on those macro concepts in Module One to influence TB control service provided by the integrated TB model are unclear, complex and unpredictable. By introducing the Module Three as a mediator variable, the conceptual framework helps health policymakers and researchers to better understand how macro concepts act on the integrated TB control health system and its different elements through determinants listed in this Module Two, further potentially influencing TB control outcomes and integrated TB model operation. Additionally, Module Three also illustrates the interaction among the blocks of the integrated TB control health system.

Notably, while this conceptual framework integrating the PRISM model can serve as a reference, health policymakers or researchers need to purposefully identify specific determinants in practical application based on their research objectives, the characteristics of TB control in the research settings or under diverse TB control systems, and data availability. We recommend adopting qualitative interviews, site surveys, secondary data analysis, and other methods to collect data to analyze the degree and path of determinants on different TB control outcomes (i.e. how and what kind of impact) mediated by the blocks of the integrated TB control health system. This will provide reliable and objective evidence for designing intervention strategies. Furthermore, given the similarity of key principles in TB control globally, four domains in the PRISM model referenced in this study may be recommended to analyze determinants of implementation in other TB control model in different settings. Methodology and result of the modifications and adjustments of the PRISM model in our framework potentially provide reference for researches in other countries in theoretical perspective at least.

(3) Design and development of intervention strategies to improve implementation of the integrated TB control model. As mentioned above, health policymakers and researchers can use the conceptual framework presented in this article to obtain evidences on the determinants of the integrated TB control model. These evidences allows them to understand problems to implementation of the integrated TB control model, the underlying causes of these problems, and the pathways by which these causes lead to problems in specific context. Consequently, our conceptual framework guides health policymakers and researchers to design and implement a series of interventions based on those evidences which would be more effectively addressing barriers to integrated TB control model operation and promote its efficiency and effectiveness in specific real-world settings, which is expected to improve the quality and sustainability of TB care.

Lastly, it is important to acknowledge that the four theories, concepts and models we integrated may overlap in terms of connotation and exposition. For example, there may be overlap between the “*comprehensive regulatory role of government*” in the New Public Service Theory and the “top-level leader” in PRISM model (23, 34, 35), as well as that between the “*value patients' demands and participation*” in the New Public Service Theory and the concept of PCC (34, 35, 39), which may lead to the ambiguity of the importance of or the entry point of understanding different concepts. However, we believe that these “overlaps” are commonly important conceptual dimensions in those theoretical models or frameworks. This study purposefully and logically integrated and synthesized those theoretical models or frameworks and so potential conflicts or redundancies could be mitigated. This approach helped us overcome the limitations of a single theoretical perspective and allowed for a comprehensive understanding and analysis of integrated TB control services.

## 5 Strength and limitations

This framework provided a multi-perspective understanding of how to deliver effective TB control care in order to achieve the global aim of ending TB, which could assist to analyze the determinants to acceptable TB care, develop “patient-centered” implementation strategies and evaluate the integrated TB control model implementation. However, there are several limitations in this study. Firstly, the development process of the framework is theoretical induction based on a synthesis of the literature, analysis and integration with existing frameworks, and the proposed theoretical guiding roles are mainly conceptual. Empirical studies need to be carried out in different contexts to validate and further modify the conceptual framework. Secondly, the conceptual framework is developed in the context where TB control service is one kind of public service provided by government. Therefore, it is suitable to analyze determinants related to TB control service provision and system operation in countries where TB control is a pubic good. Its applicability and adaptability to other countries or different TB control systems need to be further adapted and verified.

## 6 Conclusion

By integrating and synthesizing four theories, concepts and models from the fields of public management and health services, we established the conceptual framework to analyze multiple determinants of the integrated TB control services. It is expected that this conceptual framework can comprehensively and potentially serve as a reference and guide the empirical researches on integrated TB control services, such as implementation evaluation, identification of determinants, and interventions development. While this conceptual framework is currently limited to theoretically feasible and exploratory guidance, it needs empirical studies to validate and modify in the future.

## Data availability statement

The datasets presented in this study can be found in online repositories. The names of the repository/repositories and accession number(s) can be found in the article/supplementary material.

## Author contributions

XC: Data curation, Methodology, Writing – original draft. JZ: Writing – review & editing. QY: Writing – review & editing. CH: Project administration, Supervision, Writing – review & editing. YL: Conceptualization, Data curation, Funding acquisition, Methodology, Project administration, Supervision, Writing – original draft, Writing – review & editing.
